# Supplementary material for: Nutritional Counseling and Mediterranean Diet in Adrenoleukodystrophy: A Real-Life Experience
Source: Nutrients. 2024 Oct 1;16(19):3341. doi: 10.3390/nu16193341 (PMC11478612; doi:10.3390/nu16193341)
Supplement: Supplementary file 1 [file nutrients-16-03341-s001.zip › nutrients-3170236-supplementary.pdf]

Nutritional counseling and Mediterranean diet in Adrenoleukodystrophy: a real-life experience

Maria Rita Spreghini, Nicoletta Gianni, Tommaso Todisco, Cristiano Rizzo, Marco Cappa, and Melania Manco

Supplementary Table S1. *Phenotypes of X-ALD. Clinical features.*

Phenotypes of X-ALD [3,5,6-9]	Age	Clinical features
**Cerebral ALD (CC-ALD or CerALD)**	Infancy, from 2.5 years of age	The most rapidly progressive phenotype, with cognitive and behavioral impairments, due to cerebral demyelination: deterioration of academic performance, loss of ability to speak and understand, so that a misdiagnosis such as attention-deficit/hyperactivity disorder is often made. Ataxia, hemiparesis, seizures, loss of ability to walk, dysphagia, loss of visuospatial functions are the other progressive clinical features. Patients with CC-ALD often require Enteral Nutrition. Progression of this phenotype to a vegetative state or death occurs within 2-4 years.
**AdrenoMyeloNeuropathy (AMN)**	Adults (second and third decades of life)	It is characterized by ataxia and progressive paraparesis, urinary sphincter dysfunction, motor disabilities, and mild to moderate cerebral impairment due to myelopathy. Some patients present with AMN and white matter abnormalities that mimic demyelination seen in CC-ALD. Life expectancy in many cases does not differ from that of healthy individuals.
**The “Addison only” phenotype**	From 2 years of age to adulthood (average age 7.5 years)	It characterizes 10% of patients and manifests with Addison’s disease. It is caused by adrenocortical insufficiency, with endocrine symptoms occurring apparently without neurologic impairment. The

		majority of "Addison Only" phenotype displays neurologic symptoms during one's life.

Supplementary Table S2. *Non-allowed foods, according to C26:0 and total fatty acids content.*

Cereals	Meat	Meat-derived products	Fish
Breakfast cereal [16]	Bacon [19]	Bresaola [18]	Carp [18]
Flour wheat whole meal [16]	Beef [17]	Coppa [18]	Clams [18]
Pasta, whole meal wheat [16]	Chicken meat, with skin [17]	Cotechino [18]	Codfish, salted codfish [16]
Rice, whole [18]	Duck meat [18]	Ham, fat [18]	Dentex [18]
Whole meal bread [18]	Game meat [20]	Salami [18]	Eel [18]
Amaranthus [20]	Goose meat [18]	Sausages, fat [18]	Gilthead [18]
Buckwheat [18]	Lamb meat [18]	Speck [18]	Hake [18]
Corn, canned sweet corn, popcorn, corn flour (polenta), corn flakes, corn oil [16,17]	Pork meat, pork rind, fat [17]	Other cured meat rich in fat.	Herring [18]
Oat, oatmeal [18,19]	Quail meat [18]		Mackerel [17]
Quinoa [20]	Rabbit meat [18]		Mullet [18]
Rye-bread [16]	Turkey meat [16]		Mussels [18]
Spelt [20]	Brain, heart, kidney, liver from all animals, other fat meat and meat with skin [18]		Red mullet [18]
Flours from forbidden cereals			Salmon, fresh and smocked [18]
			Pilchard [17]
			Sea bass [18]
			Shrimp [16]
			Stockfish [18]
			Swordfish [19]
			Trout [18]
			Tuna fish [17]

			Yellowtail [17]
			All fish by-products (e.g. anchovy paste and tuna paste)
**Legumes**	**Egg**	**Milk and Yoghurt**	**Milk-derived products**
Beans, Borlotti [16]	Yolk [18]	Milk, whole; Milk, partially-skimmed [17]	Milk cream [18]
Broad beans, dried [18]		Yoghurt, whole milk; Yoghurt, semi skimmed milk [16,17]	Mozzarella [18]
Chick-peas [18]			Ricotta cheese [18]
Green peas [16]			Other full-fat cheese and processed cheese [18]
Lupines [18]			
Soy, soybeans and other derived-products [16,17]			
**Fruits**	**Vegetables**	**Spices**	**Nuts and seeds**
Avocado [18]	Beets [16]	Pepper, black [18]	Almonds, sweet [17]
Banana [16,17]	Broccoli [16]	Cinnamon [19]	Cashew Nuts [18]
Blueberries [16]	Cabbage [16,17]	Cumin seeds [19]	Chestnuts [18]
Coconut [18]	Dill pickle [16]	Curry [20]	Hazelnuts [18]
Grapes [16]	Green beans [16]	Ginger, ground [20]	Peanuts and Peanut butter [17]
Watermelon [16]	Lettuce [16]	Mustard [16]	Pine Nuts [18]
Olive [18]	Mushrooms, dried [19]	Oregano [19]	Pistachios [18]
Dried fruit (e.g. dried plums, dried apricots) [16]	Pumpkin [16]	Rosemary [18]	Walnuts [18]
Orange peels and lemon peels	Spinach [16]	Saffron [19]	All seeds (e.g. sesame, pumpkin seeds) [17]
	Zucchini [16,17]	Sage[18] [23]	
		Turmeric [20]	
**Fried food**	**Sweets**	**Beverages**	**Seasoning**

Chips [17]	Biscuits [16]	Milk from forbidden nuts	Butter, lard [16,17]
**All pre-cooked and pre-packaged food**	Milk Chocolate [16,17]	Juices [16]	Margarine [18]
	Cocoa and chocolate [16,17]		Olive oil, seeds oil (safflower, coconut, sunflower, corn, peanut), MCT oil [16,17]
	Ice cream [17]		Mayonnaise [16,17]
	Candies [18]		
	Torrone [18]		
	Snacks and cakes with forbidden foods		

Supplementary Table S3. *Foods with hidden fats.*

Ready-to-eat meat and fish foods	Products containing cheese and/or eggs	Products containing oil and/or milk	Products containing whole-grains cereals
Meat, canned [16]	Egg pasta [18]	Bread, made with olive oil [18]	
Fish, canned [18]		Bread, made with milk [18]	

Supplementary Table S4. *Allowed foods, according to C26:0 and total fatty acids content.*

Cereals	Meat	Meat-derived products	Fish
Pasta [18]	Beef, lean cuts, fat completely trimmed off [16]	Beef bouillon [16]	Cuttlefish (excluding tentacles) [18]
Barley, pearled, flakes, flour [18]	Chicken meat, only breast, without peel [16]	Ham, cooked and raw, lean cuts, fat completely trimmed off [16]	Dogfish [18]
Bread [16,17]	Horse meat [18]	Turkey, breast, slices [18]	Flat fish [17]

Couscous [20]	Pork meat, lean cuts, fat completely trimmed off [16,17]		Flounder [16]
Rice, rice flakes, rice [16,17]	Sausage, homemade, lean [16]		Frog [18]
Flours from permitted cereals (0 flour) [16]			Haddock [16]
			Lobster [18]
			Monkfish [19]
			Octopus [18]
			Oyster [18]
			Perch [19]
			Pike [18]
			Ray [18]
			Seabream [18]
			Sole [18]
			Squid (excluding tentacles) [18]
			Tench [18]
			Tuna fish, canned, natural, drained [16]
			Turbot [18]
**Legumes**	**Egg**	**Milk and Yoghurt**	**Milk-derived products**
Lentils, dried, peeled [18]	Egg white [16]	Milk, skimmed (0% fat) [16]	Cottage cheese 1% [16]
White beans, fresh [18]		Milk, non fat, dry, mixed with water [16]	
		Yoghurt, skimmed milk, plain (0% fat) [18]	
		Prepared foods, with skim milk (e.g. Vanilla pudding or Vanilla instant breakfast, made with skim milk) [16]	
**Fruits**	**Vegetables**	**Spices**	**Sweets**

Remove peels and seeds from permitted fruits (C26:0 content as found mainly in peel and seeds).	*Remove peels and seeds from permitted vegetables (C26:0 content as found mainly in peel and seeds).*	Basil [18]	Honey [18]
All the fruits (except the ones forbidden) [16]	Artichokes [18]	Chili pepper [18]	Jam, 100%, from permitted fruit [18]
Tropical fruit (paw paw mango, passion fruit, pineapple) [18,20]	Asparagus [18]	Parsley [18]	Sugar [18]
Allowed fruit, dried (e.g. dried apple) [16]	Carrots [16]		
	Cauliflower [18]		
	Chicory [18]		
	Cucumbers [18]		
	Eggplant [16]		
	Fennels [18]		
	Garden Cress [18]		
	Garlic [18]		
	Mushrooms, fresh [16]		
	Onions [18]		
	Peppers [16]		
	Potatoes [16]		
	Radicchio [18]		
	Tomatoes, without peel [16,17]		
	Turnips [16]		
**Beverages**	**Baking powder, Yeast, Gelatin, sheets or powder [18-20]**		
Beer [16]			
Coke [16]			
Coffee [16]			
Juices, only fruit pulp, preferably homemade [16]			
Lemonade [20]			

Lemons, juice, without peel, preferably homemade [18]			
Oranges, juice, without peel, preferably homemade [18]			
Tea [16]			
Vodka [16]			
Wine [16]			
